# Supplementary material for: Assembly dynamics of FtsZ and DamX during infection-related filamentation and division in uropathogenic E. coli
Source: Nat Commun. 2022 Jun 25;13:3648. doi: 10.1038/s41467-022-31378-1 (PMC9233674; doi:10.1038/s41467-022-31378-1)
Supplement: Supplementary file 1 — Supplementary information [file 41467_2022_31378_MOESM1_ESM.pdf]

Supplementary information for

Assembly dynamics of FtsZ and DamX during infection-related filamentation and division in uropathogenic *E. coli*

Bill Söderström*[1], Matthew J. Pittorino, Daniel O. Daley[2] and Iain G. Duggin[1].

[1] Australian Institute for Microbiology and Infection, University of Technology Sydney, Sydney, New South Wales, Australia.

[2] Dept. of Biochemistry and Biophysics, Stockholm University, Sweden.

Supplementary Figures

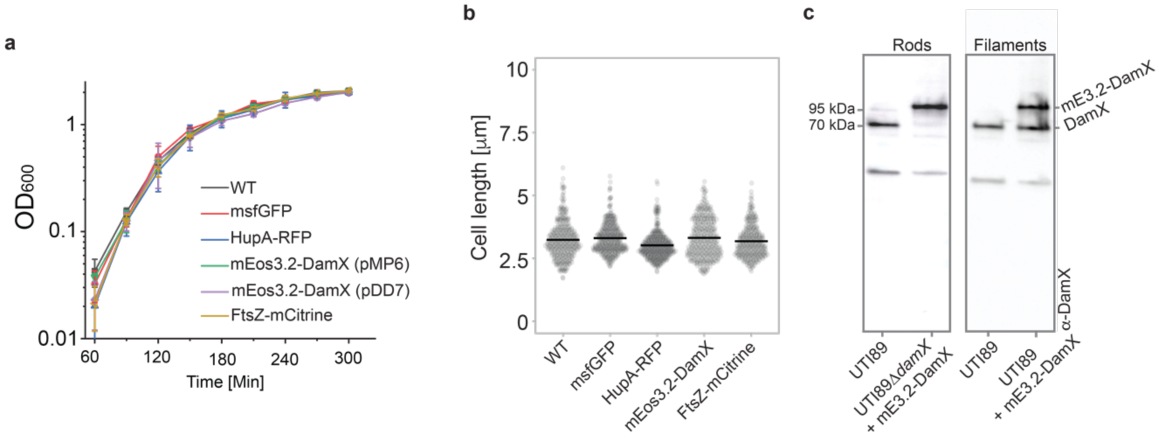

Supplementary Figure 1. Cell growth and protein detection in UTI89 containing various expression plasmids used in this study.

Cell viability measurements of strains and plasmids used in this study: WT UTI89, followed by UTI89 strains expressing msfGFP (pGI5), HupA-RFP (pSTC011) and mEos3.2-DamX (pMP6), mEos3.2-DamX (pDD7) and FtsZ-mCitrine (pHC054).

a, Growth curves. **b**, Cell lengths for the strains were: WT 3.24 ± 0.75 µm (n = 306), pGI5 3.31 ± 0.6 µm (n = 316), pSTC011 3.02 ± 0.54 µm (n = 340), and pDD7 3.32 ± 0.78 µm (n = 323). **c**, Western Blot detecting DamX protein levels in LB growth ("rods") and dispersed from the UTI model ("filaments") with and without mEos3.2-DamX (pDD7). Quantification indicated that mEos3.2-DamX was produced at 59 ± 0.05 % of total DamX levels with pDD7, calculated using the formula $\left(\frac{Int_{mEos3.2-DamX}}{Int_{mEos3.2-DamX}+Int_{DamX}}\right)$.

Measurements are from three biological replicates. Values represent Mean ± SD.

Note: mE3.2 = mEos3.2.

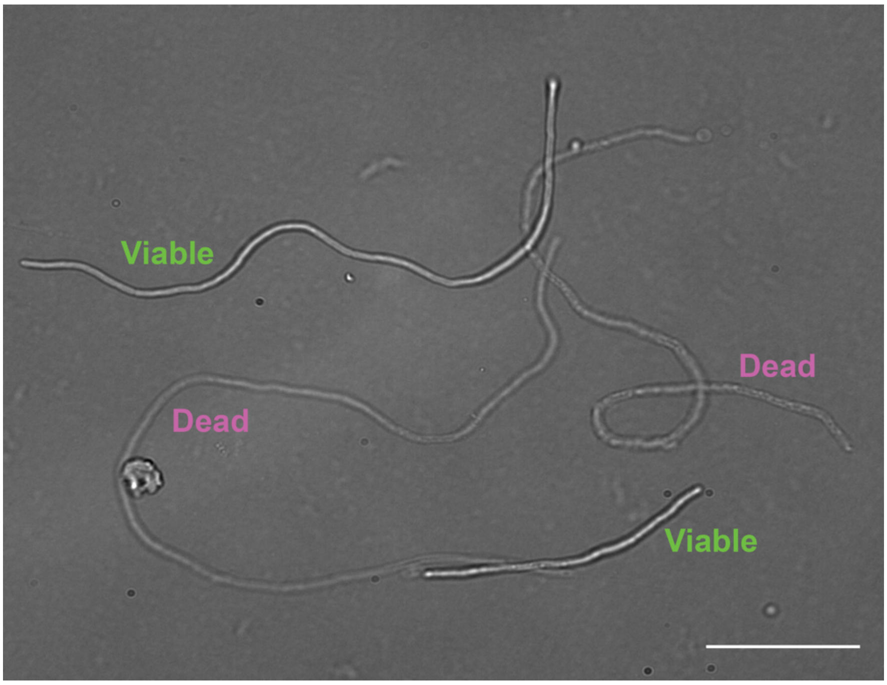

Supplementary Figure 2. WT UTI89 filaments.

Bright field image of typical filaments after infection. ‘Viable’ filaments are dense resulting in high contrast while ‘Dead’ filaments are empty membrane shells and therefore highly translucent. Scale bars = 20 μm.

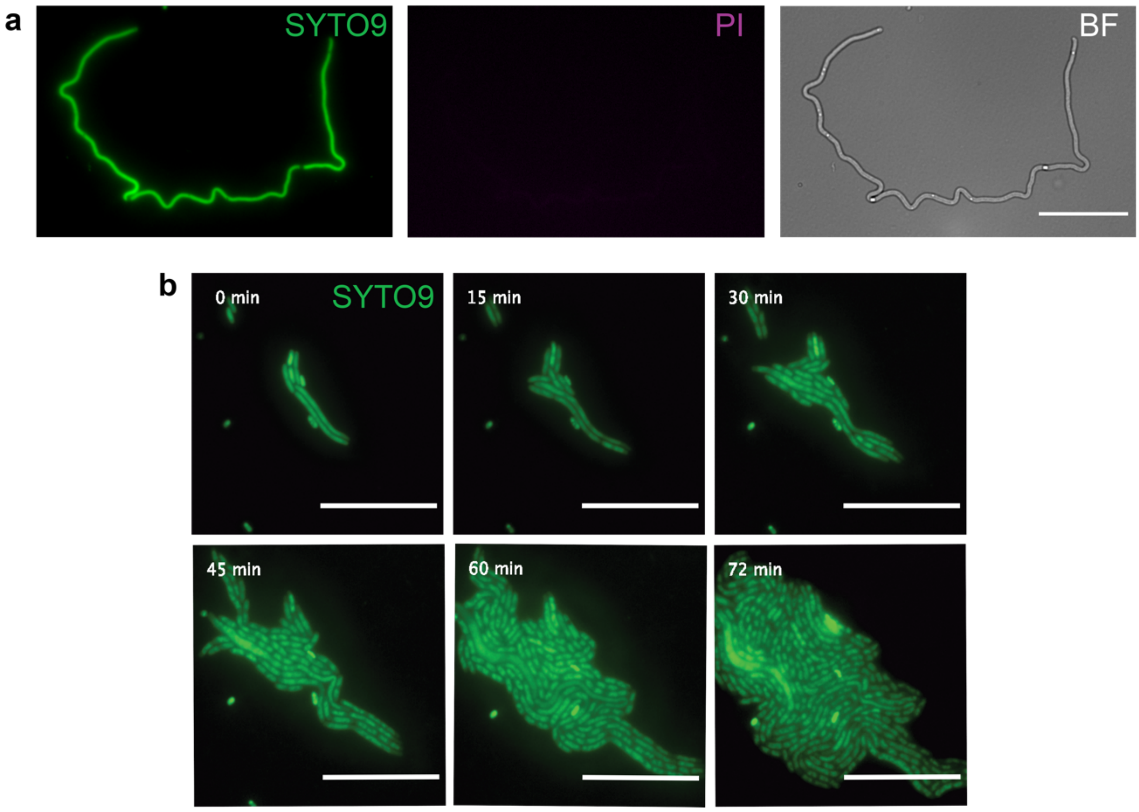

Supplementary Figure 3. Live/dead staining of UTI89 filaments.

Filaments were labelled with the *Live/dead Bac*Light staining kit containing SYTO9 and propidium iodide (PI). **a**, Filaments that are alive and show strong contrast in the bright field (BF) image only take up the SYTO9 dye (green). **b**, SYTO9 labelled filamentous and rod cells are viable, and continue dividing rapidly after staining. Scale bars = 20 μm.

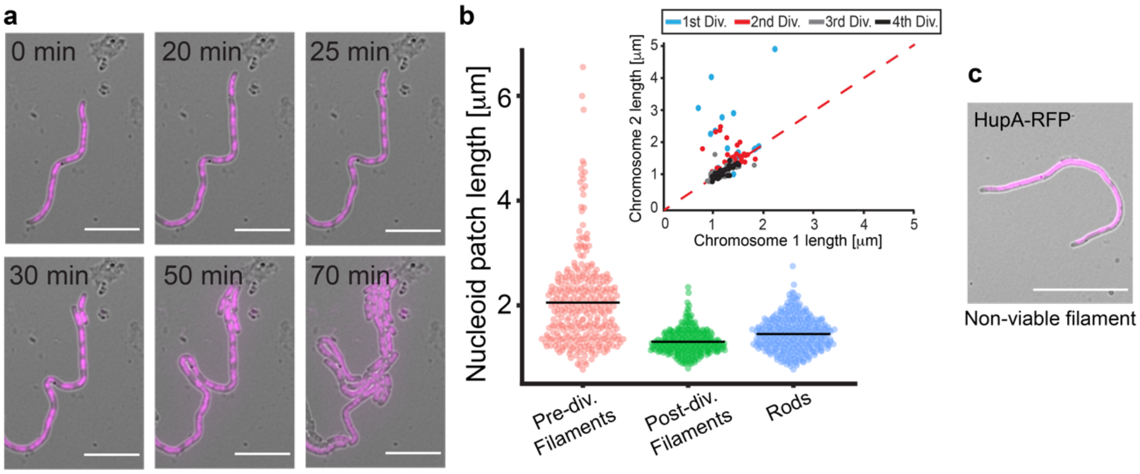

Supplementary Figure 4. Chromosome organization in filaments reverting to rods.

a, Time-lapse imaging of a representative UTI89 filament expressing HupA-RFP reverting to rods. **b**, Individual nucleoids in live filaments are longer than nucleoids in rod shaped cells. Average chromosome lengths for pre-divisional filaments were 2.05 ± 0.86 µm (red, n = 305), post-divisional filaments were 1.3 ± 0.24 µm (green, n = 254) and for rods that had not gone through an infection 1.45 ± 0.32 µm (blue, n = 308). Values represent Mean ± SD. The unpaired two-talied t-test P value was $P < 0.0001$ when comparning pre-divisional and post-divisional nucleoid patch lengths (red and green dots, respectively). Inset shows increasing symmetry of daughter chromosomes over divisions (n = 254). Blue dots represent the nucleoid lengths in the cell after the first division of a cell from a filament. Red, grey and black dots represent the nucleoids in subsequent divisions of that rod. The red striped line represents the symmetry line *i.e.*, dots on the line have equally sized chromosomes. Note that chromosome 1 was always picked as the shorter. **c**, A typical non-viable filament with HupA-RFP filling the cytoplasm. Scale bar **a** = 10 µm, **c** = 20 µm.

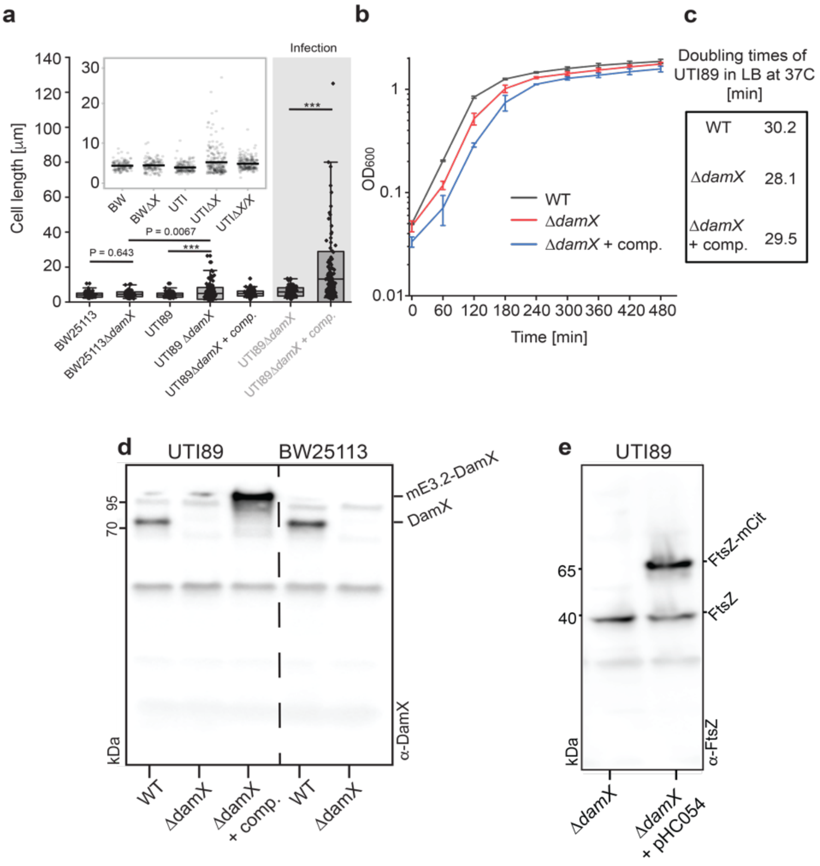

Supplementary Figure 5. Cell growth and lengths of damX-mutant strains.

a, Average lengths of various strains of BW25113 and UTI89. BW25113 = 4.38 ± 1.11 µm (n = 131), BW25113Δ*damX* = 4.45 ± 1.37 µm (n = 147), UTI89 = 3.94 ± 1.14 (n = 171), UTI89Δ*damX* = 5.25 ± 3.38 µm (n = 228) and UTI89Δ*damX* + comp. = 4.87 ± 1.41 µm (n = 219), values are mean ± S.D. Inset show magnification of cells grown in LB. Same order as in the main plot. The UTI89Δ*damX* strain was complemented with a plasmid (pMP6) constituently producing mEos3.2-DamX. Greyed out area indicate strains that were run through the infection model. Average lengths were UTI89Δ*damX* = 5.79 ± 2.35 µm (n = 197), and UTI89Δ*damX*/pMP6 = 13.17 ± 15.72 µm (n = 184). P-values are from unpaired two-tailed t-tests. *** = P < 0.0001. 95% confidence interval. Outline of boxes are S.D., midline is average. Whiskers are 99% of sample size.
b, Growth curves of UTI strains. The slight lag in growth of the UTI89Δ*damX* and UTI89Δ*damX*/pMP6 strains is possibly due to added antibiotic burden. n = 3. Error bars represent S.D. **c**, Average doubling times during log phase for the strains grown in **b**. **d**, Levels of DamX and mEos3.2-DamX (pMP6) assessed by western blotting. Average ratio between WT and mEos3.2-DamX complemented cells was 1.48, i.e., average overexpression from the plasmid was ~ 50 %. Note that both DamX and mEos3.2-DamX ran at a higher molecular weight than expected, similar as to what has previously been seen for DamX by others. **e**, Western blot showing FtsZ and FtsZ-mCitrine (pHC054) levels in UTI89Δ*damX.* All blots were done in biological triplicates.

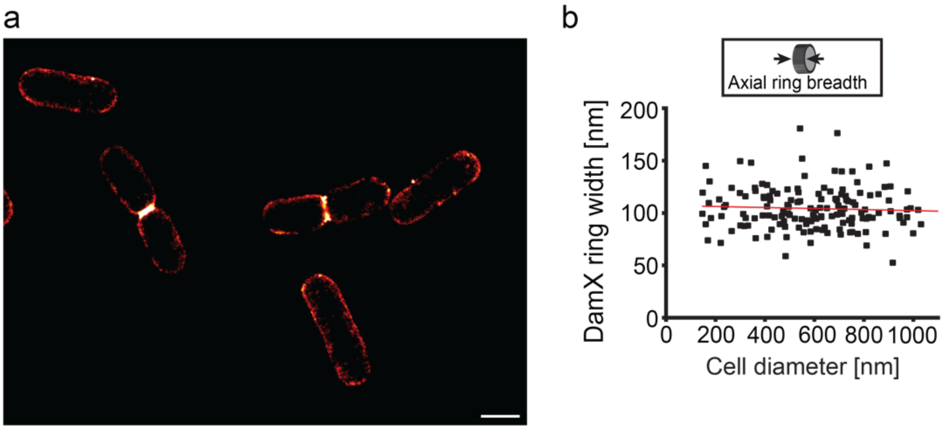

Supplementary Figure 6. mEos3.2-DamX ring widths in rods

a, PALM images of rod-shaped UTI89/pDD7 cells expressing mEos3.2-DamX. Scale bar = 1 μm. **b**, mEos3.2-DamX axial ring breadth during constriction was essentially constant with a mean of 102.5 ± 20.2 nm (n = 150). Values represent Mean ± SD. Red line represents a linear fit to the data, slope of the line was ~ -0.005. Equation of the fitted line: $y = -0.00516\ (\pm 0.00765) * x + 105.4544\ (\pm 4.64415)$

Primers used in this study:

For construction of pDD7:

Kana_out.fwd
ttgttcatgatgatatatttttatc

Kana_out.rev
tcgttccactgagcgtcagacc

AmpR_4_pAJM.011.fwd
aggggtctgacgctcagtggaacgaGTGCGCGGAACCCCTATTTGTTTAT

AmpR_4_pAJM.011.rev
gataaaaatatatcatcatgaacaaGAAGTTTTAAATCAATCTAAAGTATATATGAG

pAJM_mEos3.2.fwd
ctagagaaagaggggaaatactagatgagtgcgattaagccagacatg

mEos3.2_FtsN.rev
CTCGTTGTGCCACGAGCTCGCTTGCtcgtctggcattgtcaggcaatc

pLARGE_Fwd_FtsN (P88)
GCAAGCGAGCTCGTGGCACAACGAGATTATG

FtsN_pAJM.rev
ctcttttctggaatttggtaccgagTCAACCCCCGGCGGCGAGCCGAATG

pAJM.011.rev
ctagtatttcccctctttctctag

pAJM.011.fwd
ctcggtaccaaattccagaaaagaggc

ASEL_mEos3.2.rev
GAGCTCGCTTGCtcgtctggcattgtc

DamX.fwd
gccagacgaGCAAGCGAGCTCatgGATGAATTCAAACCAGAAGACGAGC

DamX.rev
cctcttttctggaatttggtaccgagttaCTTCAGATCGGCCTGTACCTG
For construction of pMP6:

mEos3.2.fwd:
GATAGCGCCCGGTCTAGAGGAGGTACTACC**atg**agtgcgattaagccagac

DamX.rev:
TGGCTGCAGGTCGACGGATCTTTAGGATCCttaCTTCAGATCGGCCTGTAC
