# Supplementary material for: Assembly dynamics of FtsZ and DamX during infection-related filamentation and division in uropathogenic E. coli
Source: Nat Commun. 2022 Jun 25;13:3648. doi: 10.1038/s41467-022-31378-1 (PMC9233674; doi:10.1038/s41467-022-31378-1)
Supplement: Supplementary file 3 — Description to Additional Supplementary Information [file 41467_2022_31378_MOESM3_ESM.pdf]

Descriptions of additional Supplementary Data files

Supplementary Movie 1. Upper and lower filaments: Short filaments elongating before reverting back to rods. Middle filament: initial growth before bursting. Scale bar 10 µm.

Supplementary Movie 2. A long non-viable filament. Scale bar 50 µm.

Supplementary Movie 3. Movie depicts a mixture of filaments; one non-viable long filament and multiple shorter filaments showing initial elongation before reversal. Scale bar 20 µm.

Supplementary Movie 4. Non-viable filament. Late in the movie are short rods coming from out-of-frame filament reversals observed. Scale bar 20 µm.

Supplementary Movie 5. UTI89 filaments labelled with Live/Dead stain. Green indicates live cells, magenta indicates dead cells. Scale bar 20 µm.

Supplementary Movie 6. Reversal of an UTI89 filament after a round of infection. Scale bar 10 µm.

Supplementary Movie 7. Dynamic FtsZ-mCitrine localization in a representative filament during reversal. Scale bar 20 µm.

Supplementary Movie 8. Close up of the filament shown in SM7, dynamic FtsZ-mCitrine ring assembly, disassembly and reassembly in a filament. Scale bar 2 µm.

Supplementary Movie 9. Time-lapse imaging highlighting the similarities and differences in growth between strains BW25113, BW25113ΔdamX, UTI89 and UTI89ΔdamX. Scale bar 4 µm.

Supplementary Movie 10. Left: FtsZ-mCitrine dynamics in an elongated UTI89ΔdamX cell. Right: FtsZ-mCitrine dynamics in rods as reference. Scale bar 4 µm.

Supplementary Movie 11. Reversal of a representative filament of strain UTI89ΔdamX complemented with mEos3.2- DamX (pMP6) after a round of infection. Scale bar 20 µm.

Supplementary Movie 12. Division of rod-shaped cells producing mEos3.2-DamX (pDD7) from two different infection samples. Scale bar 4 µm.

Supplementary Movie 13. De novo assembly of mEos3.2- DamX (pDD7) in a filament during reversal. Scale bar 10 µm.

Supplementary Movie 14. De novo assembly of mEos3.2- DamX (pDD7) in a filament during reversal. Scale bar 10 µm.
