# Supplementary material for: Assembly dynamics of FtsZ and DamX during infection-related filamentation and division in uropathogenic E. coli
Source: Nat Commun. 2022 Jun 25;13:3648. doi: 10.1038/s41467-022-31378-1 (PMC9233674; doi:10.1038/s41467-022-31378-1)
Supplement: Supplementary file 18 — Reporting Summary [file 41467_2022_31378_MOESM18_ESM.pdf]

nature portfolio

Corresponding author(s): Bill Söderström

Last updated by author(s): 2022/06/14

Reporting Summary

Nature Portfolio wishes to improve the reproducibility of the work that we publish. This form provides structure for consistency and transparency in reporting. For further information on Nature Portfolio policies, see our Editorial Policies and the Editorial Policy Checklist.

Statistics

For all statistical analyses, confirm that the following items are present in the figure legend, table legend, main text, or Methods section.

n/a	Confirmed	
☐	☒	The exact sample size (*n*) for each experimental group/condition, given as a discrete number and unit of measurement
☐	☒	A statement on whether measurements were taken from distinct samples or whether the same sample was measured repeatedly
☒	☐	The statistical test(s) used AND whether they are one- or two-sided<br>*Only common tests should be described solely by name; describe more complex techniques in the Methods section.*
☒	☐	A description of all covariates tested
☒	☐	A description of any assumptions or corrections, such as tests of normality and adjustment for multiple comparisons
☐	☒	A full description of the statistical parameters including central tendency (e.g. means) or other basic estimates (e.g. regression coefficient) AND variation (e.g. standard deviation) or associated estimates of uncertainty (e.g. confidence intervals)
☐	☒	For null hypothesis testing, the test statistic (e.g. *F*, *t*, *r*) with confidence intervals, effect sizes, degrees of freedom and *P* value noted<br>*Give P values as exact values whenever suitable.*
☒	☐	For Bayesian analysis, information on the choice of priors and Markov chain Monte Carlo settings
☒	☐	For hierarchical and complex designs, identification of the appropriate level for tests and full reporting of outcomes
☒	☐	Estimates of effect sizes (e.g. Cohen's *d*, Pearson's *r*), indicating how they were calculated

Our web collection on statistics for biologists contains articles on many of the points above.

Software and code

Policy information about availability of computer code

Data collection	A Nikon TiE2 with the N-STORM module was used for collecting microscopy data
Data analysis	Raw data (single-moecule data) were processed using NIS-elements (V 5.3, or ThunderSTROM (V 1.3) in Fiji/ImageJ (V 2.0.0.-rc-69/1.52n). Data and statistics were processed using Origin Pro9 (V 9.8.0.200) and GraphPad Prism 9 (V 9.3.1).

For manuscripts utilizing custom algorithms or software that are central to the research but not yet described in published literature, software must be made available to editors and reviewers. We strongly encourage code deposition in a community repository (e.g. GitHub). See the Nature Portfolio guidelines for submitting code & software for further information.

Data

Policy information about availability of data

All manuscripts must include a data availability statement. This statement should provide the following information, where applicable:

- Accession codes, unique identifiers, or web links for publicly available datasets
- A description of any restrictions on data availability
- For clinical datasets or third party data, please ensure that the statement adheres to our policy

Raw image data will be available upon request from the authors. Source data are provided with this paper.

Field-specific reporting

Please select the one below that is the best fit for your research. If you are not sure, read the appropriate sections before making your selection.

☒ Life sciences ☐ Behavioural & social sciences ☐ Ecological, evolutionary & environmental sciences

For a reference copy of the document with all sections, see nature.com/documents/nr-reporting-summary-flat.pdf

Life sciences study design

All studies must disclose on these points even when the disclosure is negative.

Sample size	Sample size for the different experiments were at least n=100. This is a commonly used number of cells examined in studies in the field.
Data exclusions	No data were excluded.
Replication	All experiments were performed in at least three biological replicas
Randomization	No randomization was needed, as all data was used
Blinding	No blinding was needed, as all data was used.

Reporting for specific materials, systems and methods

We require information from authors about some types of materials, experimental systems and methods used in many studies. Here, indicate whether each material, system or method listed is relevant to your study. If you are not sure if a list item applies to your research, read the appropriate section before selecting a response.

Materials & experimental systems

n/a	Involved in the study	
☐	☒	Antibodies
☐	☒	Eukaryotic cell lines
☒	☐	Palaeontology and archaeology
☒	☐	Animals and other organisms
☐	☒	Human research participants
☒	☐	Clinical data
☒	☐	Dual use research of concern

Methods

n/a	Involved in the study	
☒	☐	ChIP-seq
☒	☐	Flow cytometry
☒	☐	MRI-based neuroimaging

Antibodies

Antibodies used	Anitsera against DamX (Williams et al, 2013. PMID: 23290046) and FtsZ (Agrisera, cat # As10 715) were used.
Validation	This DamX antisera was a gift from Prof. David Wiess, and has been previously validated and published (PMID: 23290046). The FtsZ antibody was from Agrisera, and had has been published before (eg. Söderström et al, 2014. PMID: 24506818).

Eukaryotic cell lines

Policy information about cell lines

Cell line source(s)	DP07i bladder cells were originally derived from immortalized TEU-2 urothelial cells from an donor in accordance with the guidelines of Northwestern University's Internal Review Board of the Office for the Protection of Research Subjects, and is one of the model cell lines for UTI research. catheterized and published here PMCID: PMC100044
Authentication	The cell line was not authenticated by us.
Mycoplasma contamination	Mycoplasma testing is done quarterly in our laboratory and all tests came back negative for mycoplasma.
Commonly misidentified lines (See ICLAC register)	This is not a commonly misidentified cell line

Human research participants

Policy information about studies involving human research participants

Population characteristics	No particular exclusion of participants, except that they could have have been on any type of antibiotics for at least 6 months prior to donating urine. Both male and female participants were included in the study.
Recruitment	Volunteers could sign up on a sheet and donate urine roughly every two weeks during a six months period. All participants gave informed consent in form of a signed consent form approved by the University of Technology Sydney Human Research Ethics Committee (see below for approval number)
Ethics oversight	University of Technology Sydney Human Research Ethics Committee (HRCH REF No. 2014000452)

Note that full information on the approval of the study protocol must also be provided in the manuscript.
